# Editorial: Radiographic progression in axial spondyloarthritis

**DOI:** 10.3389/fmed.2023.1216466

**Published:** 2023-06-16

**Authors:** F. M. Pimentel-Santos, Nuh Atas

**Affiliations:** ^1^NOVA Medical School, Universidade NOVA de Lisboa, Lisboa, Portugal; ^2^Serviço de Reumatologia, Centro Hospitalar de Lisboa Ocidental, EPE, Lisboa, Portugal; ^3^Division of Rheumatology, Department of Internal Medicine, Adiyaman Education and Research Hospital, Adiyaman, Turkey

**Keywords:** axial spondylarthritis, radiographic progression, imaging, risk factors, smoking

Axial spondyloarthritis (axSpA) is a subtype of SpA that mainly affects the sacroiliac joints and the spine. One of the hallmarks of axSpA is radiographic progression, which refers to the progressive structural damage seen on imaging tests. Radiographic progression is associated with increased disability, quality of life, and higher healthcare costs (1). The data about the imaging, risk factors and treatment of radiographic progression will improve management of axSpA. This Research Topic consists of four papers—one review and three orginal articles which highlights many aspects of the radiographic progression in axSpA.

The diagnosis of axSpA is based on a combination of clinical features, laboratory tests, and imaging findings. Imaging techniques play a crucial role in detecting and monitoring the spinal and sacroiliac joint inflammation and structural damage in axSpA. Khmelinskii et al. reviewed the role of various imaging modalities in the diagnosis, classification, and monitoring of axSpA. They highlighted the advantages and limitations of X-rays, MRI scans, computed tomography (CT), and ultrasound, and discussed the use of various scoring systems, such as the modified Stoke Ankylosing Spondylitis Spine Score (mSASSS), the Berlin MRI score. They concluded that a combination of imaging tests and clinical assessments is necessary for accurate and timely diagnosis and monitoring of axSpA, and that new imaging techniques and biomarkers are promising avenues for future research.

Radiographic progression is a major concern in the management of axSpA, as it can lead to irreversible structural damage and functional impairment. Therefore, identifying the predictors of radiographic progression is an important research area. Kang et al. used group-based trajectory modelling and decision trees to identify the patterns and predictors of radiographic progression in patients with ankylosing spondylitis (AS). They classified the patients into four distinct trajectory groups based on the mSASSS scores over 10 years of follow-up: no progression, slow progression, moderate progression, and rapid progression. They found that male sex, older age, longer disease duration, elevated C-reactive protein (CRP), and higher baseline mSASSS scores were associated with a higher risk of radiographic progression. They also developed decision trees that showed the most important predictors of each trajectory group. This study provides valuable insights into the heterogeneity of radiographic progression in AS/r-axSpA and the potential for personalized risk prediction.

AxSpA disease onset is frequently observed in childbearing age. Therefore, pregnancy can affect the course and outcome of axSpA. Lee et al. investigated the impact of pregnancy on radiographic progression in 36 women with r-axSpA using CT scans of the spine and sacroiliac joints before and after pregnancy. They found that pregnancy did not significantly affect radiographic progression, as measured by the mSASSS and New York criteria scores. They also observed a trend towards decreased inflammation scores during pregnancy, suggesting a potential beneficial effect of pregnancy on disease activity. However, this study has some limitations, such as the small sample size, the lack of a control group and the imaging technique (CT) adopted.

Smoking is a well-known risk factor for radiographic progression in axSpA (2). Unlike previous studies, Nam et al. explored the impact of smoking status on radiographic progression in patients with AS/r-axSpA who received tumor necrosis factor inhibitors (TNFi) treatment. They found that current smokers had a higher risk of radiographic progression, as measured by the mSASSS scores, than former or never smokers. They also observed that current smokers had lower serum TNFi levels and higher CRP levels than non-smokers, indicating a possible mechanism for the detrimental effect of smoking on treatment response and radiographic progression. This study highlights the importance of smoking cessation in the management of axSpA.

In conclusion, radiographic progression in axSpA is a complex and multifactorial process that affects the spine and sacroiliac joints and the articles in this topic show crucial role of imaging in the diagnosis and classification, the effect of smoking and pregnancy on radiographic progression and help to predict prognosis in patients with AS. Additionally, recent studies have identified several risk factors and predictors of radiographic progression, such as male sex, older age, longer disease duration, elevated CRP, and smoking. These findings have important implications for the personalized management of axSpA and the development of new therapeutic strategies. This topic also shows more research is required and future research may focus on the validation and implementation of imaging biomarkers and scoring systems, as well as the exploration of novel treatment targets and approaches.

## Author contributions

FP-S and NA drafted the manuscript. All authors reviewed and approved the editorial.
